# Preparation and Enhanced Photocatalytic Properties of 3D Nanoarchitectural ZnO Hollow Spheres with Porous Shells

**DOI:** 10.3390/nano8090687

**Published:** 2018-09-04

**Authors:** Lan Li, Lijuan Han, Yuqi Han, Zhiwang Yang, Bitao Su, Ziqiang Lei

**Affiliations:** Key Laboratory of Eco-Environment-Related Polymer Materials, Ministry of Education of China, Key Laboratory of Polymer Materials of Gansu Province, College of Chemistry and Chemical Engineering, Northwest Normal University, No. 967 Anning East Road, Lanzhou 730070, China; lilan316@163.com (L.L.); ljhanzhang@163.com (L.H.); hexihxx@163.com (Y.H.); yjf75@163.com (Z.Y.); leizq@nwnu.edu.cn (Z.L.)

**Keywords:** ZnO, Ginkgo leaves, hollow spheres with porous shells, template-assisted two-steps method, photocatalytic property

## Abstract

By using ginkgo leaves (GL) as template and Zn(CH_3_COO)_2_∙2H_2_O as Zn source, a series of ZnO samples with special morphology were prepared via a template-assisted two-steps method without adding any other additives. The degradation of the dye MB was used to evaluate the photocatalytic property of the as-prepared samples. The results showed that when a proper amount of the template was used, a 3D nanoarchitectural ZnO hollow sphere with porous sphere shell assembled by well-distributed nanoparticles was obtained and its photocatalytic activity was much higher than that of ZnO nanoparticles. The special morphology of the sample was herein considered to be very helpful for highly efficient adsorption and activation of reactant molecules by multi-times adsorption-desorption-adsorption, efficient absorption of irradiation light by repeated absorption-reflection-absorption, and efficient separation of the photogenerated *e*^−^-*h*^+^ pairs. In addition, the formation of 3D structure of sample ZnO was also discussed.

## 1. Introduction

In recent years, environmental pollution has become one of the major problems urgently needed to be solved by society. Among the many technologies used to control environmental pollution, photocatalysis has become the future star of environmental treatment technology due to its advantages of using sunlight, complete mineralization of pollutants without secondary pollution and simple equipment. Therefore, the development of green and environment-friendly photocatalysts with higher photocatalytic activity, high stability, high solar energy utilization rate and low cost has become a hot spot and difficulty in the study of photocatalysis [1,2,3].

ZnO is one of the most widely used photocatalysts for the decomposition of organic pollutants due to its quite excellent physical, chemical, and biological features, such as wide direct bandgap (~3.37 eV), hexagonal-wurtzite crystal structure, highly robust, extremely biofriendly, low cost, and high photocatalytic property, etc. [4,5,6]. The wide bandgap of ZnO is very advantageous because UV light gets easily absorbed and afterwards the blue as well as green light is emitted corresponding to exciton and defect recombinations which is extremely helpful for photocatalytic applications [7,8,9]. In order to improve its photocatalytic activity, a number of traditional strategies have been developed, such as doping metal and nonmetal elements [10,11], depositing noble metals [12,13], coupling inorganic-inorganic and inorganic/organic semiconductors [14,15], and modification with carbon materials [16,17]. In recent years, researchers have also realized that building 3D nanoarchitecture is an efficient method for improving the performance of ZnO semiconductor [18,19,20,21]. Compared with one dimensional (1D) or two dimensional (2D) nanostructures, a three dimensional (3D) architecture with highly specific surface area will likely have unique chemical and physical properties that differ from one dimensional (1D) or two dimensional (2D) nanostructures and that lead to the enhancement of photocatalytic performance [22,23,24]. For instance, the Nirmalya group [25] prepared porous ZnO spheres via one-step growth process at low temperature by using Zn(NO_3_)_2_ as Zn source and hexamethylenetetramine (HMTA) and ammonia solution as alkaline reagents and C_6_H_5_Na_3_O_7_ as structure-directing agent, which suppressed the growth of Zn(OH)_2_ crystal in all directions and thus resulted in spherical morphology of ZnO. The as-prepared sample exhibited high photocatalytic activity for MO degradation under UV light irradiation. Yu et al. [26] obtained two kinds of 3D flower-like ZnO architectures (hydrangea and rose) by using hydrothermal method under sodium dodecyl sulfate (SDS) as surfactant and urea and HMTA as alkaline reagents. Both samples can photocatalytically degrade RhB for 25 min of UV irradiation. It can be seen that many assistant regents including precipitant, surfactants, stabilizer, slow-releasing regent and structure-directing agent and so on, are added during the related preparation processes, thus bringing about environmental pollution and higher cost. 

In fact, it can be well-understood that a special morphology of photocatalyst is certainly important for improving its photocatalytic property [27]. A heterogeneous catalytic process including the absorption of irradiated light, the adsorption and activation of the reactant molecules, and further oxidization/reduction by photogenerated *h*^+^/*e*^−^, occurs on its surface/interface. So, developing a green and low-cost method is necessary for preparing ZnO photocatalyst with special morphology and good photocatalytic activity. Template method is one of the very efficient methods for constructing 3D multilevel nanoarchitecture of the target samples because the morphology, structure, arrangement, size, and partial functional groups of the templates can play the roles of spatial confinement and structure guiding under proper conditions. In recent years, great efforts have been devoted to utilizing biomass with special structure/morphology and functional groups as templates for preparing the goal materials with special morphology and improved the performance, such as eggshells [28], butterfly wings [29], and cotton fibers [30,31,32,33].

Ginkgo is one of the oldest trees and is mainly found in the United States, China and other Asian countries. A lot of ginkgo leaves is yearly abandoned, which cause waste of resource and the pollution of environment. Its leaves are nearly semicircular and contain many constituents, such as lactones, flavonoids, polyphenol, and acids. These constituents have many functional groups, including phenolic hydroxyl and carboxyl and ketone groups, and multiple ring structure [34]. Thus, using ginkgo leaves as template to prepare ZnO with special morphology is one of efficient paths, which can not only fully use the resource but also reduce the environmental pollution. 

Our group has been interested in highly efficient photocatalysis in the long term [20,30,31,32,33,35]. In recent years, we have begun to prepare photocatalysts with multilevel nanoarchitectures and developed several template-related methods [21,30,31,32,33,36]. In this article, we report a green preparation and enhanced photocatalytic property of 3D nanoarchitectural ZnO hollow sphere with porous spherical shell via a template-assisted two-steps method by using ginkgo leaves as template. In addition, the morphology formation of the material and the approach of enhancing photocatalytic properties are also discussed. 

## 2. Materials and Methods

### 2.1. Materials

Zinc acetate dihydrate (Zn(CH_3_COO)_2_∙2H_2_O) was purchased from Shuangshuang Chemical Industry Co., Ltd., Laiyang, China.. Ethyl alcohol(EtOH) was supplied by Anhui Ante Biological Chemical Co., Ltd., Hefei, China. Methylene blue (C_1__6_H_1__8_ClN_3_S, MB) was bought from Tianjin Kaitong Chemical Reagent Co., Ltd. Tianjin, China. Tianjin Kaitong Chemical Reagent Co., Ltd. All of these reagents were analytically pure without further purification. Ginkgo leaves (GL) were collected from our campus in October. 

### 2.2. Preparation

The pretreatment of ginkgo leaves: The collected ginkgo leaves were washed with distilled water, reflux extracted by Soxhlet extractor (Beijing Xinke Laboratory Glass Instrument Co. Ltd., Beijing, China) in the mixture of water and ethanol for 16 h, and then dried naturally. The dried material was labeled as GL and stored for further use. 

The ZnO samples were prepared by a novel template-assisted two-steps method, described as follows:

First step—solve-thermal process: 3.6000 g Zn(CH_3_COO)_2_∙2H_2_O was solved in 50.0 mL EtOH and a certain amount of GL was added into the solution. Then the mixture was transferred into a 100 mL Telfon-linked autoclave, maintained at 200 °C for 10 h and then naturally cooled to room temperature, the collected solid product was dried at 60 °C for 3 h to obtain the precursor. 

Second step—calcining process: The precursor was further calcined under air atmosphere and 650 °C for 1.5 h to fully remove the GL template and to obtain goal ZnO material. To investigate the function of the template GL for the formation of special morphology and the enhancement of photocatalytic property of ZnO materials, a series of ZnO samples were prepared by changing the added amount of GL (0.0000, 0.2500, 0.8500, 1.0000, 1.5000 g), and labeled as ZnO-0,-1,-2,-3 and -4, respectively.

### 2.3. Characterization

The morphology and microstructure of the samples were examined with a field emission scanning electron microscopy (FESEM; JEOL, JSM-6701F, Tokyo, Japan) and transmission electron microscopy (TEM; JEOL, JEM-2010, Tokyo, Japan). X-ray diffraction (XRD; D/Max-2400, Tokyo, Japan) with Cu Kr radiation (*λ* = 1.5418 A) was used for phase identification. Photoluminescence (PL) spectra were measured at room temperature on a fluorescence spectrophotometer (PE, LS-55, Waltham, MA, USA) using a Xe lamp with an excitation wavelength of 325 nm. UV-Vis spectra of the samples were analyzed by Hitachi u-2001 spectrophotometer (Tokyo, Japan) from 200 nm to 800 nm. Brunauer–Emmett–Teller nitrogen adsorption-desorption experiments were carried out on the automated surface area and pore size analyzer (BET, TriStar II, Micromeritics Instrument, Atlanta, GA, USA). Solid state diffuse reflectance UV-vis spectrum was recorded at room temperature in air and spectrometer was equipped with an integrating sphere attachment; and BaSO_4_ was used as background reference materials (DRS, TU-1901, Beijing Purkinje General Instrument Co. LTD, Beijing, China).

### 2.4. Photocatalytic Activity Measurements

Photocatalytic experiments were carried out on a XPA-7 (G8) photocatalytic reactor equipped with 300 W high pressure Hg lamp (HPHL) as the UV irradiation source, which was built in the reactor and surrounded by a quartz circulating water quartz circulating water jacket to cool it, was used as the irradiation source. Catalytic reaction was carried out in a group of parallel quartz tubes. These tubes were placed to the lamp and the light distance was about 10 cm [20,30,31,32,33]. Methylene blue (MB) was used as a probe molecule to evaluate the photocatalytic activity of samples. Prior to illumination, a suspension containing 40 mg of the sample and 40 mL of 20 mg·L^−1^ MB was magnetically stirred in the dark for 30 min to attain adsorption-desorption equilibrium. The suspension was then illuminated, 5 mL of the suspension was sampled at regular intervals and centrifuged to remove the photocatalyst and the absorbance value of the supernatant was measured at 664 nm (*λ*_max_ of MB). The decolorization efficiency and first-order kinetic equation of MB solution are *D_t_*% = (*A*_0_ − *A_t_*)/*A*_0_ × 100% and ln(*C*_0_/*C*_t_) = *k*_1_ *t*, in which *A*_0_ and *A_t_*, *C*_0_ and *C_t_* are the absorbance and concentration values of MB solution at the initial time *t* = 0 and reaction time *t*, respectively, *k*_1_ is the pseudo-first-order reaction rate constant (min^−1^).

## 3. Results and Discussion

### 3.1. XRD Analysis

**Figure 1:**
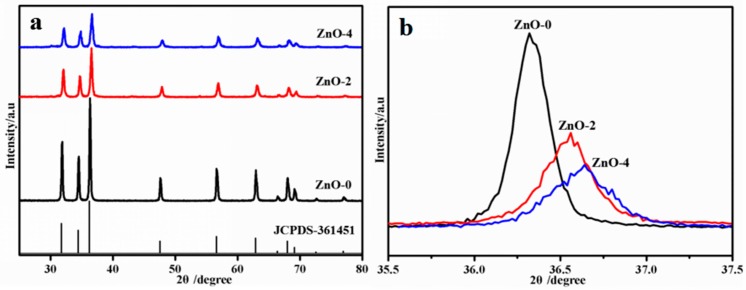
XRD patterns (**a**) and the local amplification (**b**) of the samples ZnO-0, -2 and -4.

The XRD patterns of the samples ZnO-0,-2 and -4 are depicted in Figure 1. It can be found that these samples are wurtzite ZnO, well matching with JCPDS card no. 361451, but their diffraction intensities are different and the intensity order is ZnO-0 > ZnO-2 > ZnO-4. And from Figure 1b, it can be also seen that their peaks successively occur shift to higher diffraction angle (2*θ*), which means the decrease of ZnO crystal cell, and become broaden. The phenomena are related to many factors, such as the lattice defect, crystalline degree, and size of the samples. The particles size of the samples, calculated from Scherrer’s equation, are 52, 30 and 28 nm, respectively. So the as-obtained materials are nanosized and the template GL can efficiently control the size of the goal material. The crystalline property of the samples can be understood as follows. On the one hand, when the amount of the added GL is relatively low, the amount of the adsorbed-Zn^2+^ ions on its surface is high on the unit surface of the template and a large amount of ZnO nucleus is produced during the solvothermal process, which is benefit to the growth of the ZnO crystal. When the amount of the added GL is relatively high, the amount of the adsorbed-Zn^2+^ ions is low and thus the amount of ZnO nucleus is low, which is disadvantageous for the growth of the ZnO crystal. On the other hand, because the combustion of the template GL and the conversion of adsorbed Zn^2+^ ions to ZnO all need [O] during the calcining process in air atmosphere. The more the amount of used GL is, the more the needed [O] is. So, in the obtained ZnO samples with the presence of the template GL, the existence of oxygen vacancy might be expected and brings about the crystalline decrease of the samples. 

### 3.2. SEM Analysis

**Figure 2:**
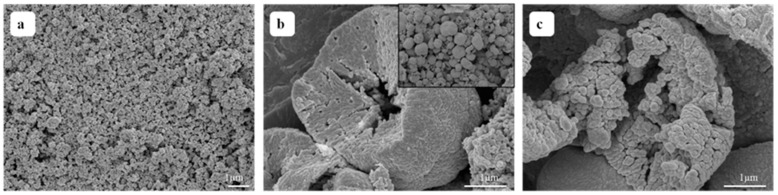
SEM images of the samples ZnO-0 (**a**); -2 (**b**) and -4 (**c**).

FESEM images of the samples ZnO-0,-2 and -4 are shown in Figure 2. ZnO-0 (Figure 2a), obtained without introducing GL, is a nanoparticle aggregate. ZnO-2 (Figure 2b), obtained by using a proper amount (0.8500 g) of GL, is basically hollow spheres, and the size of these spheres is different (the inset of Figure 2b). Herein the 3D nanoarchitectural ZnO hollow spheres are called multi-structured material because the shell of the hollow spheres is assembled by nanoparticles. Compared with ZnO-2, ZnO-4 (Figure 2c) shows irregular morphology, which results from the collapse of hollow sphere due to its thin sphere shell.

**Scheme 1:**
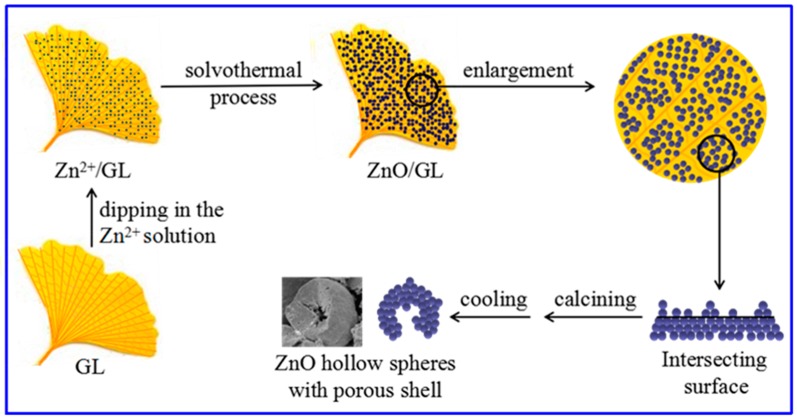
The formation processes of 3D nanoarchitectural ZnO hollow spheres with porous shells.

GL has many veins and mesophylls surrounded by veins and the size of the mesophylls is different and the closer the mesophyll is to the petiole, the smaller the mesophyll is (see in Scheme 1). So, it is easily understood that the size of the mesophylls controls the size of as-obtained ZnO hollow spheres and the smaller the mesophyll is and the smaller the size of as-obtained ZnO spheres is. And with increasing the amount of added GL (more than an optimal one), the sphere shell becomes thinner and thinner and will partially/fully collapse during the cooling process and thus the multi-structure is partially/fully destroyed.

### 3.3. TEM Analysis

**Figure 3:**
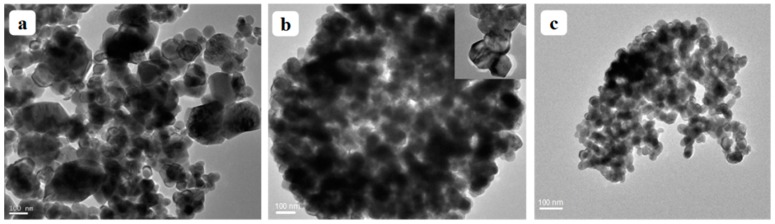
TEM images of the samples ZnO-0 (**a**); -2 (**b**) and -4 (**c**).

The detailed structures of the samples ZnO-0, ZnO-2 and ZnO-4 are observed by their TEM images. The sample ZnO-0 is nanoparticles with wide size distribution (40–300 nm) and obvious aggregation is observed. The sample ZnO-2, prepared by introducing an appropriate amount GL as template, is a structure of hollow sphere, its porous sphere shell is assembled by nanoparticles of almost mono-size (about 30 nm) and regular hexagon (seen in the inset of Figure 3b). However, the sample ZnO-4 is of heavily broken hemisphere (Figure 3c). 

The SEM and TEM results of these samples are confirmed each other and demonstrate that a proper amount of GL can play a role of the template for efficiently controlling the morphology/microstructure and further improving the property of the goal material (seen in Section 3.4). 

On the formation of the 3D nanoarchitectural ZnO hollow spheres with porous shells, it can be described as follows: Two sides (up-down) structures of the ginkgo leaves are different. On the up-side, the arrangement of the cells is relatively order and dense. At the first step of preparation—the solvothermal process, Zn^2+^ ions are firstly adsorbed on the two sides of GL, the amount of adsorbed Zn^2+^ ions is different on the two sides, and thus the amount and density of the in-situ formed ZnO nanoparticles is also different (seen in Scheme 1). At the second step (calcining process), the ZnO/GL precursor is calcined under air atmosphere and a proper temperature to remove the template GL by its combustion and then cooled naturally. During the natural cooling, the curling phenomenon occurs because of different stress of the both sides and thus the multilevel structure is formed. It can be expected that when the amount of the GL is lower than a proper value, the as-obtained sample should be a certain number of hollow spheres with relative dense sphere shell while the sample is composed a large amount of badly broken hemispheres and nanoparticles when the amount of the GL is much higher than a proper value. So, adding a proper amount of GL can ensure the formation of the special morphology, hollow spheres with porous shells.

### 3.4. BET analysis

**Figure 4:**
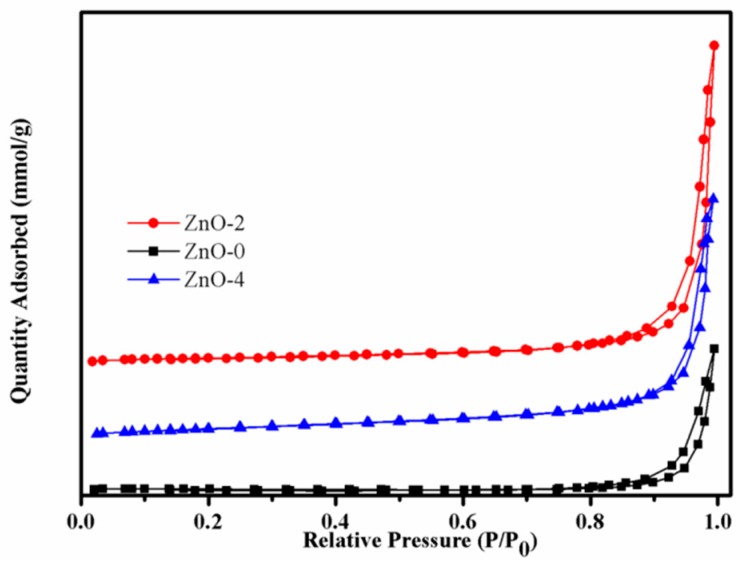
Nitrogen absorption–desorption isotherms of the samples ZnO-0, -2 and -4.

Nitrogen adsorption/desorption measurements were used to test the Brunauer−Emmett−Teller (BET) surface areas of the as-prepared samples. The BET surface areas of samples were investigated by nitrogen adsorption-desorption experiments. As shown in Figure 4. All the samples exhibit typical type-III curves. The quantity adsorbed of ZnO prepared by GL showed higher than ZnO-0. 3D nanoarchitectural ZnO hollow spheres having high specific surface area was obtained.

### 3.5. DRS Analysis

**Figure 5:**
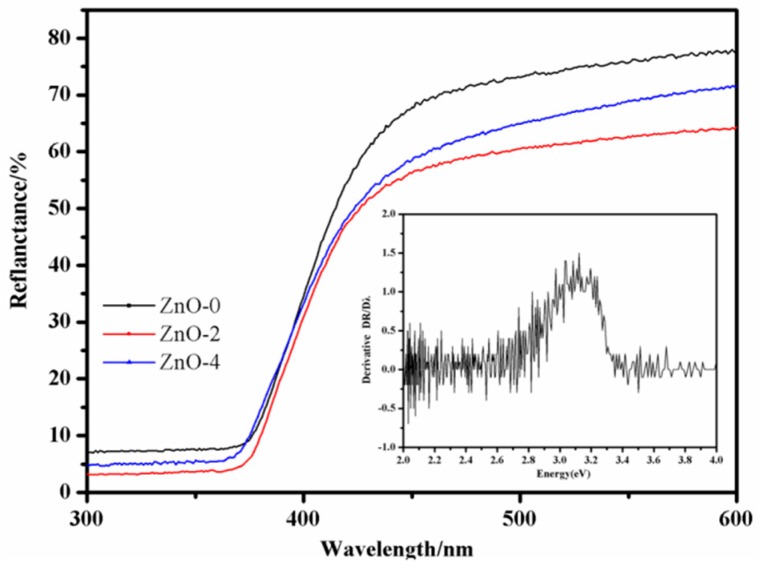
Diffuse reflectance measurement of the samples ZnO-0, -2 and -4, together with the first derivative of ZnO-2 (inset) to describe the band gap.

Figure 5 shows the UV-Vis DRS spectra of samples. The band gap energy (*Eg*) values of samples were calculated as approximately 3.20 eV from the first derivative of the reflectance to a wavelength (*DR/Dλ*, *R* is reflectance and *λ* is wavelength), which were the lower than bulk ZnO NPs powder (*Eg* = 3.37 eV) [4]. This variation in band gap might be due to various factors such as structural parameter, carrier concentration, grain size, and induced defects. 

### 3.6. Photocatalytic Activity

**Figure 6:**
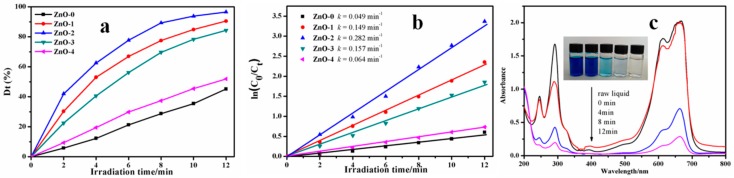
(**a**) *D*_t_ ~ *t* of the samples ZnO-0~4; (**b**) The pseudo-first-order reaction rate constant of the samples ZnO-0~4; (**c**) Absorbance spectra for photocatalytic degradation of MB on the sample ZnO-2.

Based on the unique structural and morphological characteristic of ZnO, the photocatalytic activity for MB degradation is tested. Figure 6a shows the degradation rate of MB on the samples ZnO-0~4. And all the samples exhibit very high photocatalytic performance for the degradation of MB solution. Herein it is much meaningful that the activities of the samples ZnO-1~4 prepared with the presence of GL are higher than ZnO-0 which is prepared without the presence of GL. The photocatalytic activity firstly increases with increasing the amount of added GL and reaches an optimal activity when the amount is about 0.8500 g and then decreases with further increasing the GL amount. Figure 6b gives the results of ln (*C*_0_/*C*_t_)~*t*. It is obvious that the photocatalytic degradations of MB solution apparently for all samples follow first-order kinetic behavior ln(*C*_0_/*C*_t_) = *k*_1_ *t*. The reaction rate constant *k*_1_ can be obtained from the Figure 6b. For example, the photocatalytic activity of ZnO-2 (0.282 min^−1^) is nearly 6 times as ZnO-0 (0.049 min^−1^). Figure 6c shows that the absorbance spectra of the MB solutions in the range 200–800 nm under regular interval of time in order to verify the mineralization ability of the sample ZnO-2. It shows the decreasing trend of absorption for ZnO as photocatalyst. A strong absorption band at 664 nm represents maximum wavelength for MB dye. In dark conditions, the decrease of absorbance was equal to about 2%. Due to adsorption of dye molecules, ZnO as photocatalyst plays a vital role for photo-degradation application. Decrease in absorbance intensity clearly confirms that ZnO are acting as photocatalyst for the degradation of dye. Complete degradation of dye was observed in 12 min under UV irradiation, the color of MB solution changes from dark blue to colorless. 

From XRD, SEM and TEM results of the samples, many factors, such as the size, crystalline degree, defect/oxygen vacancy, and morphology of the samples, are considered to affect the photocatalytic performance. In general, for a heterogeneous photocatalytic reaction, the smaller the size of the used catalyst is, the larger its surface area is, the more the active site is, and the higher its photocatalytic activity is. And it is well known that a proper crystallinity and some structural defect of a catalyst are also important for ensuring its good catalytic property. 

**Figure 7:**
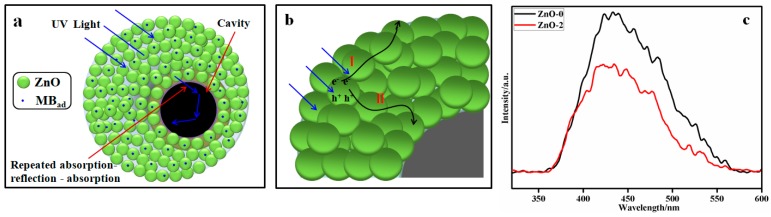
(**a**) Schematic drawing of highly efficient adsorption of the reactant molecules and high efficient absorption of irradiation light in the ZnO hollow sphere with porous sphere shell; (**b**) Schematic illustration for the migration process of the photogenerated electron in the sphere shell; (**c**) The photoluminescence spectra (PL) of the samples ZnO-0 and ZnO-2.

For the highest activity of the sample ZnO-2, the special morphology/microstructure is herein considered to be a more important factor (Figure 2 and Figure 3). Why the 3D nanoarchitecture of a photocatalyst is so important to its good photocatalytic performance? We consider that on the enhanced photocatalytic performance of as-obtained ZnO hollow spheres with porous shells, the special nanoarchitecture structure of the material herein plays many roles of ensuring higher efficient adsorption of the reactant molecules by multi-times adsorbing-desorbing-re-adsorbing, higher efficient absorption of the irradiation light by repeatedly absorbing-reflecting-absorbing through the porous shell and inner cavity of the hollow sphere (seen in Figure 7a), and efficient separation of photogenerated *e*^−^-*h*^+^ pairs (seen in Figure 7b) for enhancing the photocatalytic performance. PL technique is a powerful tool to demonstrate the separation efficiency of photogenerated charges. The lower the PL intensity of a photocatalyst, the lower the recombination of *e*^−^-*h*^+^ pairs is, the higher its photocatalytic activity is [32]. Figure 7c illustrates the PL spectra of the samples ZnO-0 and ZnO-2 with an excitation wavelength of 325 nm. It can be seen that there are several weak emission peaks for the sample ZnO-0. These origin broad unstructured emissions bands in ZnO cover the blue, green, yellow, orange, and red spectral zones, and they are frequently assigned to native defects such as oxygen vacancies (*V*_O_), zinc vacancies (*V*_Zn_), oxygen interstitials (*O*_i_), zinc interstitials (*Zn*_i_), and their complexes. In general, the strong PL means high recombination of photogenerated carriers, suggesting the low photocatalytic activity [37,38,39]. The PL intensity of the sample ZnO-2 is remarkably weaker than that of ZnO-0, which indicates fast separation of photogenerated *e^−^/h^+^* pairs in ZnO-2. The efficient interparticle transfer of the photogenerated charges through the grain boundaries has been suggested and demonstrated [40,41]. In this paper, we suggest a more efficient separation path of the *e^−^/h^+^* pairs in the described-above special morphology of ZnO photocatalyst by comparing the activities and morphologies of the sample ZnO-2 and ZnO-0 and ZnO-4 (seen in Figure 3). We suggest that the potential energy (μ_e_) of the photogenerated e^−^ should be different on the irradiated and no irradiated ZnO particles and μ_e_ is higher on the irradiated ZnO particles than on no irradiated ones. So, the migration of these photogenerated electrons is easier (seen in Figure 7b(I)) from the irradiated ZnO particles to no irradiated ones than among the irradiated ZnO particles (seen in Figure 7b(II)). Therefore, it brings about fast separation of the photogenerated *e^−^/h^+^* pairs and increases the stability of photogenerated holes h^+^ and thus further increases the photocatalytic activity of the special 3D sample.

## 4. Conclusions

A 3D nanoarchitectural ZnO sample, hollow spheres with porous shellss, was successfully prepared via template-assisted two-steps method by adding a proper amount of the GL template without adding any other additives. The special structure of the GL template and preparing method play an important role in the formation of the sample with special morphology. 3D nanoarchitectural ZnO hollow spheres with porous shells show excellent photocatalytic property for the degradation of MB molecules and its excellent photocatalytic property is considered to be mainly ascribed to highly efficient adsorption of reactant molecules by multi-times adsorption-desorption-adsorption and absorption for the reached light by repeated absorption-reflection-absorption through its porous shells and inner cavity and separation of the photogenerated *e*^−^-*h*^+^ pairs by the migration of photogenerated electrons by interparticle transfer, especially from the irradiated ZnO particles to no irradiated ones in the special morphology of hollow spheres with porous shellssll.
